# Analytical and Clinical Validation for RT-qPCR Detection of SARS-CoV-2 Without RNA Extraction

**DOI:** 10.3389/fmed.2020.567572

**Published:** 2020-10-15

**Authors:** José P. Miranda, Javiera Osorio, Mauricio Videla, Gladys Angel, Rossana Camponovo, Marcela Henríquez-Henríquez

**Affiliations:** ^1^ELSA Clinical Laboratory, IntegraMedica, part of Bupa, Providencia, Santiago, Chile; ^2^Advanced Center for Chronic Diseases (ACCDiS), Pontificia Universidad Católica de Chile & Universidad de Chile, Santiago, Chile; ^3^Department of Nutrition, Diabetes, and Metabolism, School of Medicine, Pontificia Universidad Católica de Chile, Santiago, Chile; ^4^School of Medicine, Pontificia Universidad Católica de Chile, Santiago, Chile; ^5^Millennium Nucleus in Cardiovascular Magnetic Resonance, Millennium Science Initiative Program, ANID, Santiago, Chile

**Keywords:** SARS-CoV-2, COVID-19, diagnostic, direct RT-qPCR, RNA extraction, pandemic (COVID-19)

## Abstract

**Background:** The recent COVID-19 pandemic has posed an unprecedented challenge to laboratory diagnosis, based on the amplification of SARS-CoV-2 RNA. With global contagion figures exceeding 4 million persons, the shortage of reagents for RNA extraction represents a bottleneck for testing globally. We present the validation results for an RT-qPCR protocol without prior RNA extraction. Due to its simplicity, this protocol is suitable for widespread application in resource-limited settings.

**Methods:** Optimal direct protocol was selected by comparing RT-qPCR performance under a set of thermal (65, 70, and 95° for 5, 10, and 30 min) and amplification conditions (3 or 3.5 uL loading volume; 2 commercial RT-qPCR kits with a limit of detection below 10 copies/reaction) in nasopharyngeal swabs stored at 4°C in sterile Weise's buffer pH 7.2. The selected protocol was evaluated for classification concordance with a standard protocol (automated RNA extraction) in 130 routine samples and 50 historical samples with Cq values near to the clinical decision limit.

**Results:** Optimal selected conditions for direct protocol were: thermal shock at 70°C for 10 min, loading 3.5 ul in the RT-qPCR. Prospective evaluation in 130 routine samples showed a 100% classification concordance with the standard protocol. The evaluation in historical samples, selected because their Cqs were at the clinical decision limit, showed 94% concordance with our confirmatory standard, which includes manual RNA extraction.

**Conclusions :** Our results validate the use of this direct RT-qPCR protocol as a safe alternative for SARS-CoV-2 diagnosis in the case of a shortage of reagents for RNA extraction, with minimal clinical impact.

## Introduction

In late 2002, an epidemic outbreak of severe acute respiratory syndrome (SARS) was described in China's Guangdong province, and its cause was attributed months later to the SARS-CoV coronavirus (1–3). According to statistics from the World Health Organization (WHO), this outbreak reached 26 countries, with an estimated number of cases of 8096, of which 774 died (4).

A new strain of coronavirus (SARS-CoV-2), causing COVID-19 disease, was reported in December 2019 in Wuhan, Hubei province, China (5). Since then, this outbreak has spread globally, forcing the WHO to decree a pandemic on March 11, 2020, when the number of confirmed cases reached 118,000 within 114 countries (6). After 2 months of this decree, many countries found themselves with strict quarantine policies and several confirmed cases that globally rose to 5.7 million, while death due to COVID-19 reached 357,736 (WHO; May 29th, 2020; https://covid19.who.int/).

The rapid availability of the complete genome of this new virus, submitted on January 5 to GeneBank under the access code MN908947 (7) and released by January 12, allowed for the development of specific primers to amplify the genetic material of SARS-CoV-2 in order to diagnose patients with COVID-19, using the reverse transcription quantitative real-time polymerase chain reaction (RT-qPCR) technique. Until May 9, 2020, the Foundation for Innovation in New Diagnostics (FIND; http://www.finddx.org) listed on its website at least 141 different commercially available diagnostic kits for nucleic acid amplification tests (NAAT) for SARS-CoV-2 with CE-IVD certification, and this number increased to 314 kits when considering other types of certification for clinical diagnosis. NAAT-based kits, the standard analysis for early detection of SARS-CoV-2 (8), share characteristics in their processing, including (1) sample collection typically performed with nasopharyngeal, or oropharyngeal swabs, (2) RNA extraction, and (3) reverse transcription of RNA, PCR amplification, and detection.

Due to the tremendous number of tests that are being carried out globally, reagents necessary for the SARS-CoV-2 detection process are scarce, especially those required for RNA extraction, which represents a dangerous bottleneck when ensuring the rapid diagnostic procedure for patients with COVID-19 and its appropriate clinical management (9, 10). This work aimed to develop and validate a protocol for the detection of SARS-CoV-2 based on RT-qPCR without RNA extraction. The widespread validation and use of this kind of protocol might contribute to ensuring diagnostic continuity in the current setting of globally limited resources for manual and automatic viral RNA extraction, helping to control the outbreak, aid in characterization, and vigilance.

## Materials and Methods

### Clinical Specimens

Clinical samples for the standardization and validation experiments were obtained from the routine of ELSA Clinical Laboratories, IntegraMedica, part of Bupa, Santiago, Chile. This laboratory serves 1 million patients annually, with more than 12 million total tests, and corresponds to the biggest private laboratory provider in Chile. Sampling was performed using nasopharyngeal swabs in symptomatic patients and then stored at 4°C in tubes containing sterile potassium sodium phosphate buffer (Weise's buffer) pH 7.2 (Merck, Cat. No.109468), until analysis.

All procedures followed were in accordance with the Helsinki Declaration. Before sampling, all patients requesting SARS-CoV-2 RT-qPCR testing were asked to approve and sign consent forms allowing for the use of their anonymized samples and clinical information for epidemiological vigilance and research.

### Standard RT-qPCR Protocol

For the standard protocol, routinely used in the laboratory for the detection of SARS-CoV-2, an aliquot of 180 ul of the sample from the nasopharyngeal swab, including 10 ul of extraction control, was used to extract RNA with the MagNA Pure 96 DNA and Viral NA LV Kit (Roche Diagnostics, Cat. No. 06374891001) in the MagNA Pure 96 System (Roche Diagnostics). Then, 10 ul of the extracted RNA was used for amplification by RT-qPCR using the LightMix® Modular Wuhan CoV RdRP-gene kit (Roche, Cat. No. 53-0777-96) in a Cobas z 480 system (Roche Diagnostics). This kit allowed for the amplification of a 100 bp fragment from a conserved region of the RNA-dependent RNA polymerase (RdRP) gene and was used as a reference for standardization, validation, and final evaluation in problematic samples. Positive, negative, and blank controls (no template or no RT enzyme) were included in all the amplification procedures. The analytical sensitivity reported by the provider was 10 copies/reaction. Automatic analysis was performed using LightCycler® 480 Software, Version 1.5.

### Direct RT-qPCR Protocol Standardization

For the standardization of the direct SARS-CoV-2 detection protocol without RNA extraction steps, 50 ul aliquots from the primary sample (nasopharyngeal swabs) of 5 anonymized patients were subjected to heat shock (65, 70, or 95°C) during different incubation times (5, 10, or 30 min), and then were quickly placed at 4°C until the moment of amplification. From the sample subjected to heat shock, two different loading volumes were used for the RT-qPCR (3 or 3.5 ul of the sample). The later sample-treatment was evaluated using the LightMix® Modular Wuhan CoV RdRP-gene and the SARS-CoV-2/SARS-CoV Multiplex REAL-TIME PCR Detection Kit (DNA-Technology, Cat. No. R3-P436-23/9EU R3-P436-S3/9EU), using the respective controls, following the manufacturer's instructions, and loaded in a Roche Cobas z 480 or a DTlite thermal cycler (DNA-Technology), respectively. The multiplex of the DNA-Technology kit amplifies three targets; the first is general to SARS-CoV-like coronaviruses (CoV-like); the other two targets are specific to SARS-CoV-2, for E gene (CoV-2 E) and N gene (CoV-2 N). The analytical sensitivity reported by the provider was 10 copies/reaction. Automatic analysis was performed using the DTmaster software for the DNA-Technology kit, included in the DTlite system. The cycle of quantification (Cq) values obtained with each kit and condition were compared with those obtained with the standard protocol for the same samples.

### Validation of the Direct RT-qPCR Protocol in Routine Clinical Samples

For the validation stage, results obtained for the described sample-treatment conditions and amplification conditions were evaluated by an expert board in the laboratory to select the set of conditions (direct RT-qPCR protocol) with the best performance, considering Cq-value for positive samples and clinical/analytical classification concordance. The following direct RT-qPCR protocol was selected for further validation: heat shock at 70°C for 10 min and then quickly placed at 4°C, loading 3.5 ul of the sample for RT-qPCR amplification with the SARS-CoV-2/SARS-CoV Multiplex REAL-TIME PCR Detection Kit.

This protocol was further evaluated for (a) repeatability and analytical variability of Cqs, using an abbreviated protocol that included four anonymized clinical samples run in triplicates, (b) statistical and clinical equivalence of the obtained Cqs for positive samples in comparison with the standard protocol already in use (*n* = 27) and (c) clinical classification concordance with the standard protocol already in use (*n* = 130).

### Evaluation of the Validated Protocol Using Problematic Samples

To test the performance of the direct RT-qPCR protocol in samples with Cqs near or beyond the discriminatory value (Cq ≥40) as by our standard protocol (denominated “problematic samples” in this text for simplicity), we analyzed 50 historical samples with this condition in parallel by the direct and standard RT-qPCR protocols. In our laboratory routine, we set a confirmation algorithm for these samples (Supplementary Figure 1), where RNA was manually extracted using an RNeasy Mini Kit (Qiagen, Cat. No. 74106), and RT-qPCR amplification was re-run for samples with 1 ng/ul of RNA or more after manual RNA extraction, to establish a final classification. For samples with <1 ng/ul RNA after manual RNA extraction, patients were contacted to take a new sample. Results for the comparison between the direct RT-qPCR protocol, the standard laboratory protocol (with automatic RNA extraction), and the confirmatory protocol (with manual RNA extraction) are presented.

### Statistics

Descriptive statistics, statistical analyzes, and Bland-Altman graphs for the comparison of the different protocols were performed using Stata MP 14.2.

## Results

Results from the standardization experiment are shown in Table 1. Based on these results and the expert laboratory board (MHH, MV, GA, JO), we established the following direct RT-qPCR protocol as optimal for further validation:

1. Obtain an aliquot of 50 ul from the primary sample, stored at 4°C in Weise's buffer
2. Thermal shock at 70°C for 10 min
3. Store at 4°C until loading the sample into a plate
4. Perform the RT-qPCR using 3.5 ul of the sample with the SARS-CoV-2/SARS-CoV Multiplex REAL-TIME PCR Detection Kit (high sensibility kit).

**Table 1:** Result of the standardization of optimal conditions for direct RT-qPCR.

**Temperature (°C)**	**–**	**65°**	**65°**	**70°**	**70°**	**95°**	**95°**		**65°**	**65°**	**70°**	**70°**	**95°**	**95°**
**Incubation time (min)**		**30**	**30**	**10**	**10**	**5**	**5**		**30**	**30**	**10**	**10**	**5**	**5**
**Sample volume (ul)**		**3**	**3.5**	**3**	**3.5**	**3**	**3.5**		**3**	**3.5**	**3**	**3.5**	**3**	**3.5**
	**Standard protocol**	**LightMix® Modular Wuhan CoV RdRP-gene**						**SARS-CoV-2/SARS-CoV Multiplex RT-qPCR**						
**Anonymized sample**	**Cq**	**Cq**						**Target**	**Cq**					
								CoV-like	36.6	36.4	36.5	**36.3**	37.6	37.1
4,794	35.8	38.6	40.0	38.1	**39.0**	39.5	38.7	CoV-2 E	36.7	36.5	36.6	**36.3**	37.7	37.2
								CoV-2 N	36.7	37.1	36.7	**36.7**	38.5	37.7
								CoV-like	17.4	17.2	17.4	**17.3**	22.6	23.6
4,793	16.2	24.5	24.5	24.1	**23.2**	21.6	21.8	CoV-2 E	17.5	17.2	17.5	**17.4**	22.6	23.7
								CoV-2 N	17.7	17.6	18	**17.8**	22.8	23.7
								CoV-like	(–)	(–)	(–)	**(–)**	(–)	(–)
3,023	(–)	(–)	(–)	(–)	**(–)**	(–)	(–)	CoV-2 E	(–)	(–)	(–)	**(–)**	(–)	(–)
								CoV-2 N	(–)	(–)	(–)	**(–)**	(–)	(–)
								CoV-like	(–)	(–)	(–)	**(–)**	(–)	(–)
1,929	>40	(–)	(–)	(–)	**(–)**	(–)	(–)	CoV-2 E	(–)	(–)	(–)	**(–)**	(–)	(–)
								CoV-2 N	(–)	(–)	(–)	**(–)**	(–)	(–)
								CoV-like	35.7	31.6	31.7	**31.6**	33.5	33.3
2,980	30.1	33.9	33.5	33.3	**33.7**	32.9	32.7	CoV-2 E	35.8	31.8	31.7	**31.7**	33.7	33.3
								CoV-2 N	36.0	31.7	31.7	**31.7**	33.7	33.1

*Different treatment conditions were evaluated with two commercial kits and compared with the standard protocol. Amplification on negative or blank samples was not detected. In bold are the optimal conditions selected for the direct protocol*.

**Figure 1:**
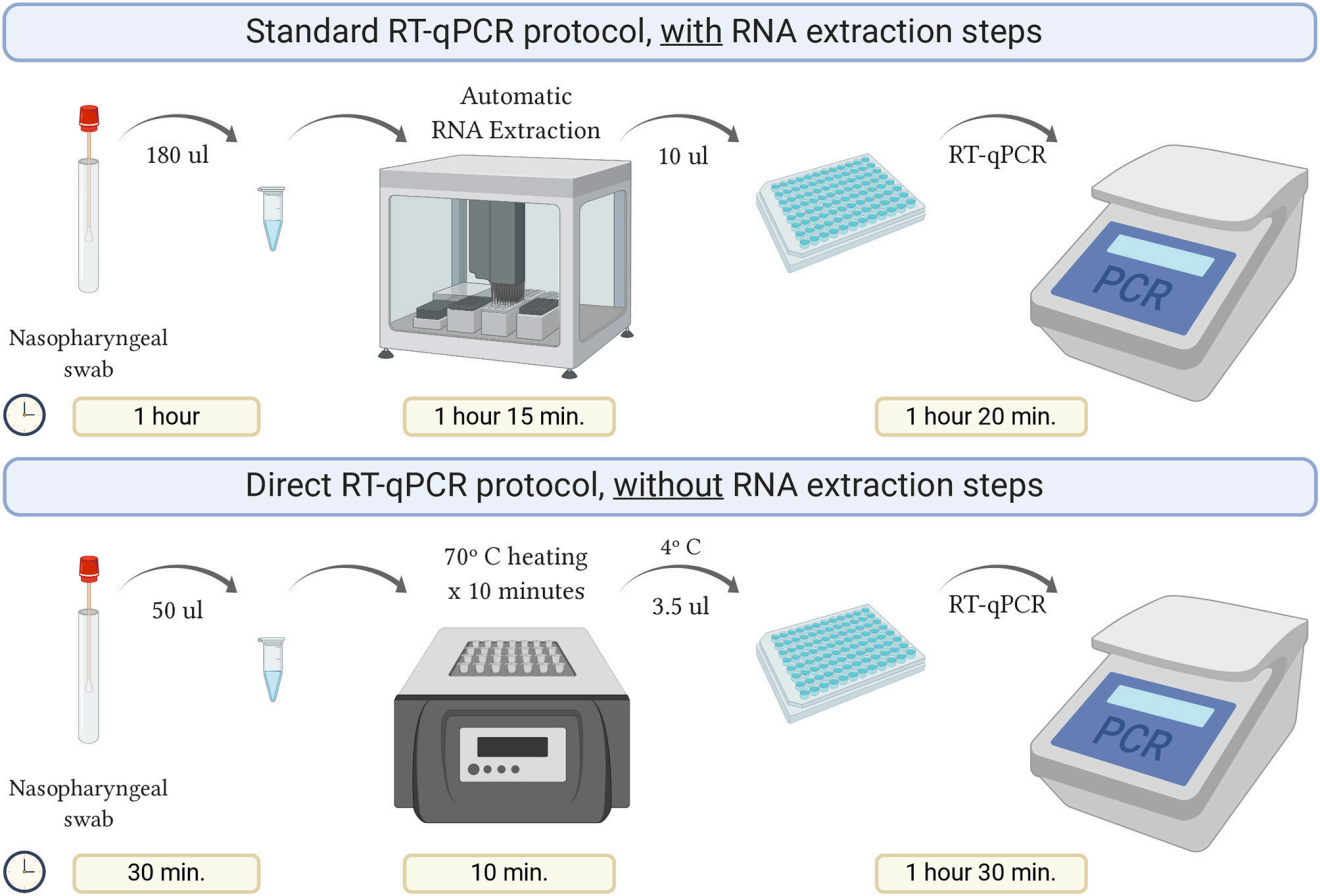
Schematic representation of the direct RT-qPCR protocol without RNA extraction steps, compared to the standard protocol. Created with BioRender.com.

A summary of this procedure and a comparison with the standard protocol is detailed in Figure 1.

Using the direct protocol saved about 40% of the analysis time compared to the standard, which allowed for enhanced daily processing capability.

Table 2 resumes the results of a brief repeatability assay to characterize the variability of the Cqs roughly. Note that Cqs present a very low intra-assay variability; therefore, we proceeded to the validation stage.

**Table 2:** Repeatability and pre-validation of selected RT-qPCR protocol conditions in four clinical samples.

	**Standard protocol**	**SARS-CoV-2/SARS-CoV Multiplex RT-qPCR**				
**Anonymized sample**	**Cq**	**Target**	**Cq1**	**Cq2**	**Cq3**	**Informed diagnostic**
		CoV-like	(–)	(–)	(–)	
6,608	(–)	CoV-2 E	(–)	(–)	(–)	Negative
		CoV-2 N	(–)	(–)	(–)	
		CoV-like	28.1	27.4	27.4	
3,407	26.5	CoV-2 E	28.1	27.3	27.4	Positive
		CoV-2 N	27.6	27.1	27.2	
		CoV-like	36.1	35.2	37.4	
3,380	34.3	CoV-2 E	36.1	35.3	37.3	Positive
		CoV-2 N	36.5	35.0	37.6	
		CoV-like	34.3	35.2	34.5	
3,420	37.6	CoV-2 E	34.4	35.2	34.6	Positive
		CoV-2 N	34.3	35.5	34.9	

*Incubation time 10 min, temperature 70°C, sample volume 3.5 ul using a DNA-Technology kit*.

**Figure 2:**
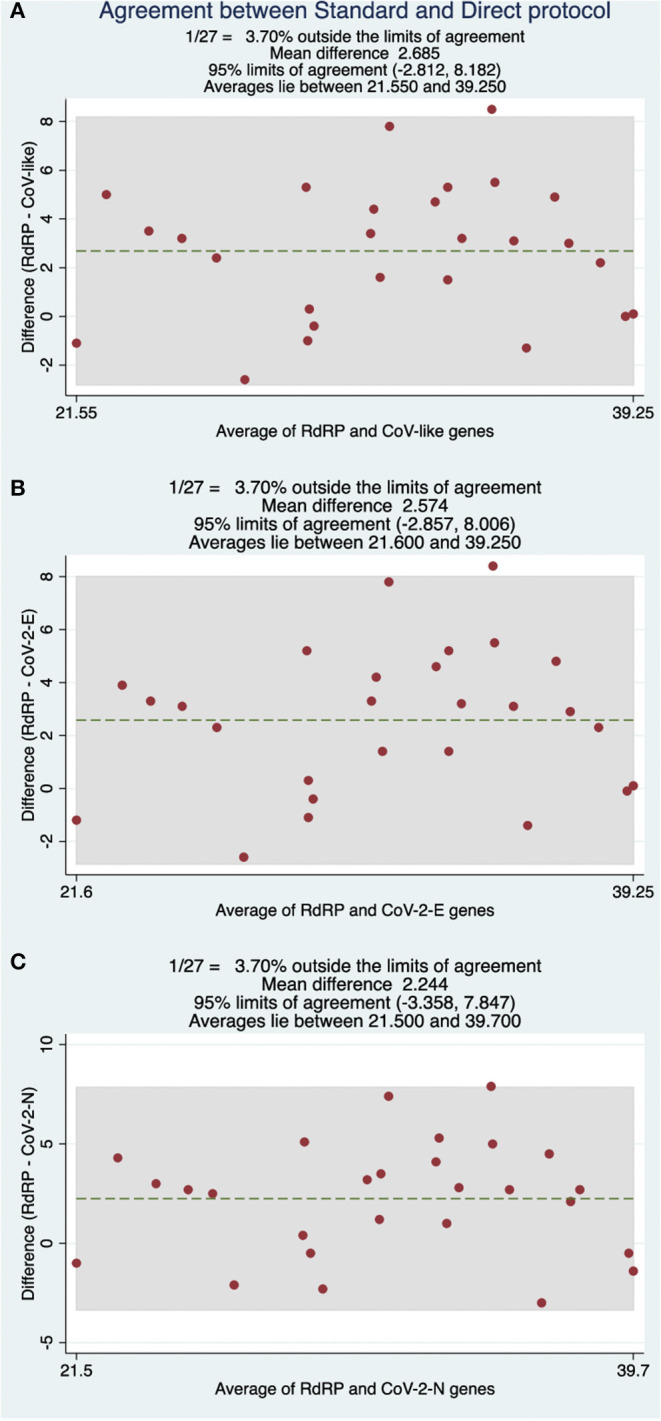
Bland-Altman comparisons of Cq values obtained between the RdRP gene under standard protocol and **(A)** CoV-like, **(B)** CoV-2 E-gene, and **(C)** CoV-2 N-gene using a DNA-Technology kit with the direct protocol.

Diagnostic classification concordance was then evaluated in 130 routine samples, ran prospectively using the direct RT-qPCR protocol and the standard protocol. We found full concordance with 27 positive patients for SARS-CoV-2 (Table 3). Moreover, the same was found for the other 103 patients, with negative confirmation by both methods. Based on these results, we established that the performance of the direct protocol was very high, with neither false positive nor false negative results in the 130 samples analyzed, thus yielding 100% concordance (**Table 5**). Additionally, we found that Cqs for positive samples were significantly lower when using the direct protocol and DNA-Technology kit. The median Cq for the standard protocol was 34.1, while for the direct protocol, it was 30.4 (CoV-like), 30.6 (CoV-2 E gene), and 30.7 (CoV-2 N gene) (*P* < 0.0009 for each, Wilcoxon signed-rank test) (Table 3). In order to visualize this difference, a Bland-Altman graph was made using the standard protocol as a reference (RdRP gene) (Figure 2). This graph also shows that, in general, there is a good concordance between the standard and the direct protocol, with only one sample slightly outside the limits of agreement and, besides, this graph confirms that for each gene of the direct protocol (DNA-Technology kit), the Cq necessary for the classification of the sample was lower than for the standard protocol.

**Table 3:** Validation of the direct RT-qPCR.

	**Standard protocol**	**SARS-CoV-2/SARS-CoV Multiplex RT-qPCR**			**Informed diagnostic**	
**Anonymized positive samples**	**Cq RdRP**	**Cq CoV-like**	**Cq CoV-2 E**	**Cq CoV-2 N**	**Standard protocol**	**Direct RT-qPCR**
3,775	28.4	29.4	29.5	30.7	Positive	Positive
3,787	25.0	20.0	21.1	20.7	Positive	Positive
3,793	32.6	29.2	29.3	29.4	Positive	Positive
3,795	39.0	30.5	30.6	31.1	Positive	Positive
3,798	29.1	28.8	28.8	28.7	Positive	Positive
3,809	26.5	23.3	23.4	23.8	Positive	Positive
3,810	21.0	22.1	22.2	22.0	Positive	Positive
3,811	37.6	32.1	32.1	32.6	Positive	Positive
3,814	34.1	32.6	32.7	33.1	Positive	Positive
3,820	27.2	24.8	24.9	24.7	Positive	Positive
3,823	35.3	30.6	30.7	31.2	Positive	Positive
3,824	36.0	30.7	30.8	30.7	Positive	Positive
3,826	35.4	32.2	32.2	32.6	Positive	Positive
3,841	32.0	30.4	30.6	30.8	Positive	Positive
3,843	25.6	22.1	22.3	22.6	Positive	Positive
3,844	25.6	28.2	28.2	27.7	Positive	Positive
3,846	35.2	36.5	36.6	38.2	Positive	Positive
3,852	28.9	29.3	29.3	29.4	Positive	Positive
3,854	31.5	26.2	26.3	26.4	Positive	Positive
3,855	35.4	27.6	27.6	28.0	Positive	Positive
3,859	33.2	28.8	29.0	29.7	Positive	Positive
3,865	37.0	33.9	33.9	34.3	Positive	Positive
4,890	39.0	39.0	39.1	40.4	Positive	Positive
5,003	39.2	34.3	34.4	34.7	Positive	Positive
5,174	38.7	35.7	35.8	36.6	Positive	Positive
9,107	39.3	37.1	37.0	36.6	Positive	Positive
13,848	39.3	39.2	39.2	39.8	Positive	Positive
Median Cq (IQR)	34.1 (28.4–37.6)	30.4 (27.6–33.9)	30.6 (27.6–33.9)	30.7 (27.7–34.3)		
*P*-value	–	0.0002^*^	0.0003^*^	0.0009^*^		

* *Wilcoxon signed-rank test, Cq values from the standard protocol were used as a reference. IQR, interquartile range*.

*We evaluated 130 clinical cases, of which 27 were positive (20.8% positivity). Negative samples were omitted from this table*.

With the purpose of evaluating the performance of direct RT-qPCR protocol in samples with Cq close to and over the discriminatory value, a total of 50 historical samples analyzed with the standard protocol and confirmed by manual extraction according to our current quality assurance algorithm, were re-analyzed by direct RT-qPCR (Table 4). As observed, there is a high classification agreement with the confirmatory protocol that reaches 94% (Table 5), which is also observed in terms of Cq (Table 4). The only discordance was found for two samples classified as positive with the direct RT-qPCR protocol (possibly false positive), while they showed amplification over cycle 40 when they were processed by automatic and manual RNA extraction with the standard protocol. We also obtained a sample with amplification after cycle 40 with the direct method, previously classified as positive according to the standard method (possibly false negative). In every case, direct RT-qPCR presents amplification close to cycle 40, so it is plausible that the sample corresponded to patients with a viral load very close to the detection limit for both protocols.

**Table 4:** Analysis of concordance in the classification of 50 problematic historical samples, which were re-processed using the direct RT-PCR validated protocol.

	**Standard protocol**	**Standard protocol (manual extraction)**	**Direct SARS-CoV-2/SARS-COV multiplex RT-qPCR**			**Informed diagnostic**	
**Anonymized samples**	**Cq RdRP**	**Cq RdRP**	**Cq CoV-like**	**Cq CoV-2 E**	**Cq CoV-2 N**	**Standard protocol (manual)**	**Direct RT-qPCR**
604	>40	>40	(–)	40.3	39.2	Negative	Negative
189	>40	34.3	38.3	38.6	38.4	Positive	Positive
193	>40	33.0	36.0	36.0	36.3	Positive	Positive
605	>40	34.1	32.4	32.4	32.9	Positive	Positive
624	>40	>40	40.4	40.3	39.2	Negative	Negative
650	>40	>40	36.1	36.1	36.9	**Negative**	**Positive**
727	>40	>40	39.7	38.9	39.8	Negative	Negative
1,265	>40	36.3	35.9	35.9	36.7	Positive	Positive
1,287	>40	28.6	26.7	26.8	27.2	Positive	Positive
1,288	>40	>40	(–)	(–)	(–)	Negative	Negative
1,298	>40	26.5	25.7	25.5	25.0	Positive	Positive
1,309	>40	29.9	28.2	28.1	28.7	Positive	Positive
1,346	>40	28.5	28.2	28.3	28.3	Positive	Positive
1,421	>40	(–)	38.9	38.9	(–)	Negative	Negative
1,433	>40	30.0	28.9	28.8	30.1	Positive	Positive
1,434	>40	37.0	35.3	35.3	36.7	Positive	Positive
1,576	>40	31.3	29.2	29.1	29.4	Positive	Positive
1,637	>40	(–)	(–)	(–)	(–)	Negative	Negative
1,684	>40	30.8	29.5	29.5	30.4	Positive	Positive
1,708	>40	34.9	33.3	33.3	34.5	Positive	Positive
1,726	>40	(–)	(–)	(–)	(–)	Negative	Negative
1,734	>40	36.1	33.9	33.9	35.0	Positive	Positive
1,742	>40	33.9	32.4	32.4	32.3	Positive	Positive
1,792	>40	(–)	(–)	(–)	(–)	Negative	Negative
1,802	>40	(–)	(–)	(–)	(–)	Negative	Negative
1,850	>40	24.9	24.2	24.4	25.0	Positive	Positive
1,883	>40	34.0	32.6	32.6	32.7	Positive	Positive
1,921	>40	32.9	32.3	32.3	31.8	Positive	Positive
1,929	>40	>40	37.9	37.8	38.2	**Negative**	**Positive**
1,968	>40	30.2	28.4	28.4	29.6	Positive	Positive
1,983	>40	30.6	29.1	29.2	29.7	Positive	Positive
1,997	>40	(–)	(–)	40.9	(–)	Negative	Negative
3,440	>40	29.3	33.7	33.8	33.4	Positive	Positive
3,445	>40	35.5	36.1	36.2	36.7	Positive	Positive
3,479	>40	31.1	31.7	31.8	32.0	Positive	Positive
3,484	>40	(–)	(–)	(–)	(–)	Negative	Negative
3,507	>40	(–)	(–)	(–)	(–)	Negative	Negative
3,539	>40	34.7	35.9	36.0	35.9	Positive	Positive
3,587	>40	>40	(–)	(–)	41.4	Negative	Negative
3,602	>40	35.6	39.9	39.8	38.6	Positive	Positive
3,654	>40	(–)	(–)	(–)	(–)	Negative	Negative
4,167	>40	35.9	37.9	37.9	38.6	Positive	Positive
4,168	>40	29.8	37.4	37.5	37.8	Positive	Positive
4,169	>40	34.4	35.5	35.6	36.0	Positive	Positive
4,170	>40	33.5	36.5	36.5	36.5	Positive	Positive
4,174	>40	(–)	(–)	(–)	(–)	Negative	Negative
4,183	>40	>40	(–)	(–)	(–)	Negative	Negative
7,226	>40	38.0	41.9	42.0	46.4	**Positive**	**Negative**
7,626	>40	30.5	33.6	33.7	33.8	Positive	Positive
8,259	>40	37.3	37.7	38.5	38.1	Positive	Positive

*For standard protocol, classification was performed using results from manual RNA extraction. Differences in the diagnostic classification between the standard protocol and the direct protocol are in‘bold*.

**Table 5:** Evaluation of the clinical performance of direct RT-qPCR protocol compared to the standard protocol as reference.

	**Validation stage (Cq <40)**	**Evaluation of problematic samples (Cq ≥40)**
True positive	27	31
True negative	103	16
False positive	0	2
False negative	0	1
Total	130	50
Classification concordance	100%	94%

*Concordance for each, the validation and evaluation of problematic samples are shown*.

## Discussion

The evidence presented allows for validating the direct RT-qPCR protocol as a comparable alternative to the standard protocol in routine for the detection of SARS-CoV-2. Consequently, the clinical impact of replacing the standard protocol currently in use in our laboratory with the new direct RT-qPCR protocol is estimated to be minimal, given the high classification agreement between both techniques, without false negatives and false positives in a total of 130 samples analyzed by both methods as part of the described validation stage.

In a small number of samples using the standard protocol in our laboratory routine, sharp amplification was obtained after cycle 40. We have denominated these samples as “problematic” because it is difficult to establish whether it is a true or a false negative. In our experience with the routine use of the Roche LightMix® Modular Wuhan CoV RdRP-gene kit associated with automatic nucleic acid extraction in the MagNA Pure 96 system, ~64% of the abovementioned samples (which should be reported as negative using the standard protocol) changed their classification when repeated by manual extraction (Table 4), evidencing the loss of sensitivity in cases with low viral load when performing nucleic acid extraction on an automated platform. Consequently, we evaluated the direct protocol in 50 samples in this situation and found 94% of concordance with the confirmatory protocol (manual extraction followed by amplification using the LightMix® Modular Wuhan CoV RdRP-gene kit) as the reference, with only two possible false positive results and one possible false negative result for the direct RT-qPCR. Even when these results were far superior to results obtained by our standard protocol, we concluded that the direct protocol should be evaluated with caution when there is amplification near to the detection limit and thus, we decided to set a confirmatory protocol for samples with Cq >37 by the direct method and to re-analyze these samples using manual RNA extraction and a different RT-qPCR kit (Supplementary Figure 1).

The demand for tests for the detection of SARS-CoV-2 by RT-qPCR grows every day around the world to allow for the management of COVID-19 disease. Despite the high number of commercially available NAA tests, the process requires various reagents and supplies that have created the bottleneck for rapid analysis. Among these reagents, undoubtedly, the most scarce have been those related to the extraction of the genetic material from the coronavirus. In order to cope with this problem, many research laboratories have joined in the duty of analyzing clinical samples from patients in several countries, playing a fundamental role in maintaining the diagnostic process and containing the spread of the coronavirus, notwithstanding the stocks that are reserved in these centers are also limited and do not allow for the long-term diagnostic process. Taking this background into account, we believe that it is vital that clinical laboratories take an active role in generating knowledge to maintain the diagnostic process and to share this knowledge so that others can also implement it.

In the present work, we successfully validated a protocol for performing RT-qPCR bypassing the initial nucleic acid extraction step with a high classification agreement with our standard protocol. So far, a protocol with similar conditions for sample treatment can be found in the literature (11), nonetheless, researchers have found that its use required on average 6.1 (±1.6) more cycles to reach the diagnosis classification, which is not optimal for the service in the clinical laboratory due to the susceptibility of false negatives near to the detection limit. The amplification kit used in their protocol was the LightMix® Modular Wuhan CoV E-gene (Roche Diagnostics), which has a reported analytic sensibility of 10 copies/reaction or fewer, performance in agreement with an independent research (12). Considering these authors also used a high sensitivity kit, we believe, therefore, that components of the medium used for swab storage (VTM) could have influenced the displacement of the number of cycles required for the diagnosis. In our protocol, samples were stored in Weise's buffer, which is an aqueous solution of sodium hydrogen phosphate (Na_2_HPO_4)_ and potassium dihydrogen phosphate (KH_2_PO_4_). This buffer allows for low interference with RT-qPCR due to the lack of other interfering salts such as sodium chloride in PBS buffer or BSA in the VTM medium. Moreover, our selected amplification kit was manufactured by DNA-Technology and also reported an analytical sensitivity of 10 copies/reaction, which agrees with the high classification performance we obtained.

Finally, we considered whether our protocol was extendable to other kits, including those with intermediate sensitivity. With this question in mind, we used the Genesig® kit (Cat No. Z-Path-2019-nCoV, analytical sensitivity <100 copies/reaction) on 58 samples. The optimal conditions for direct RT-qPCR protocol, in this case, were different, with heat shock at 95°C for 5 min and loading a sample of 6.5 ul. With this protocol, we obtained 23 positive and 35 negative samples, all with a correct diagnostic classification when compared with our Roche standard. The median value for Cq obtained by Roche was 27.2 (IQR 24.3–33.8), while for the Genesig® kit, this value was significantly lower, with a median of 25.0 (IQR 24.0–30.8), *P* = 0.002, Wilcoxon signed-rank test (Data not shown).

Considering these data, we believe that one of the most critical factors for the success of a direct protocol is to use a sample storage buffer that does not interfere with the RT-qPCR reaction. Furthermore, it strongly suggests that the optimal conditions for use with the direct protocol must be previously tested for each kit.

For the study presented, we considered that the most significant limitation was associated with our inability to evaluate a greater number of tests available on the market. Including them could have made it possible to develop a more robust and extensible protocol. We also believe that it would be interesting to evaluate our theory that the sample storage buffer is essential in the success of the direct RT-qPCR technique. Although our results and previous publications suggest the relevance of the storage buffer, new analyzes are required to deepen and conclude on the optimal conditions and formulations for the operation of the technique we describe.

Despite the fact that this protocol allows clinicians to reduce the processing time considerably, we believe that its implementation should be restricted only to those clinical laboratories in which the lack of RNA extraction reagents is a limiting factor when complying with the diagnostic process. This suggestion is made because the most important object is to ensure the quality of analysis in the diagnosis of patients. Consequently, we hope that the use of this protocol will contribute to ensuring the diagnostic process of COVID-19.

## Data Availability Statement

All datasets presented in this study are included in the article/Supplementary Material.

## Ethics Statement

The studies involving human participants were reviewed and approved by IntegraMedica ethics committee. The patients/participants provided their written informed consent to participate in this study.

## Author Contributions

JM wrote the manuscript with the help of MH-H. JM, JO, MV, and GA developed the analysis of samples and statistics. RC contributed to the interpretation of the results. MH-H conceived the study and was in charge of the direction and planning. All the aforementioned authors have made a substantial contribution to the development of this work.

## Conflict of Interest

The authors declare that the research was conducted in the absence of any commercial or financial relationships that could be construed as a potential conflict of interest.
