# Supplementary material for: Analytical and Clinical Validation for RT-qPCR Detection of SARS-CoV-2 Without RNA Extraction
Source: Front Med (Lausanne). 2020 Oct 15;7:567572. doi: 10.3389/fmed.2020.567572 (PMC7593567; doi:10.3389/fmed.2020.567572)
Supplement: Supplementary file 1 [file Image_1.PDF]

Supplementary Figure 1. General sample processing algorithm. Workflow for SARS-CoV-2 sample detection according to the Cq values obtained by RT-qPCR, both for the standard and for the direct protocol. Created with BioRender.com

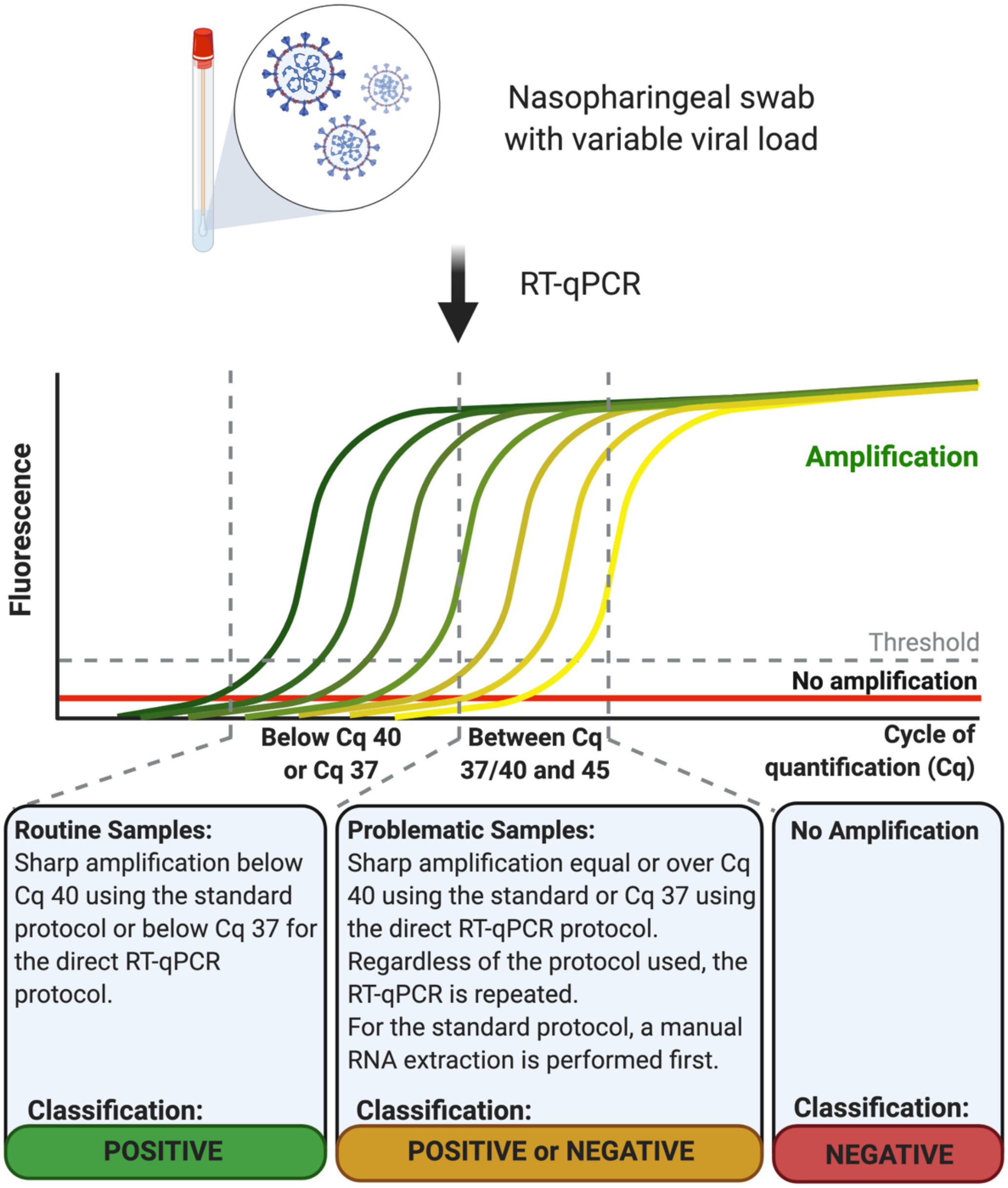

Supplementary Figure 1 describes the routine processing and classification of SARS-CoV-2 samples in the laboratory. Samples with sharp amplification below Cq 40 with standard protocol or Cq 37 with direct protocol, are classified as positive for SARS-CoV-2. For those samples with sharp amplification equal or over Cq 40 (standard protocol) / Cq 37 (direct protocol) and less than Cq 45, the RT-qPCR is repeated, considering a manual extraction step for the standard protocol. The classification in these problematic samples is positive or negative for SARS-CoV-2 depending on the result obtained in the confirmatory RT-qPCR. Samples that did not exceed the detection threshold are classified as negative.
